# Pre-hospital lung ultrasound for cardiac heart failure and COPD: is it worthwhile?

**DOI:** 10.1186/s13089-018-0104-5

**Published:** 2018-09-10

**Authors:** Mirko Zanatta, Piero Benato, Sigilfredo De Battisti, Concetta Pirozzi, Renato Ippolito, Vito Cianci

**Affiliations:** Emergency Department of Arzignano Hospital, AULSS 8 Berica, Via Parco 1, 36071 Arzignano, Vicenza Italy

**Keywords:** Pre-hospital, Lung, Ultrasound, Dyspnea, Heart failure, COPD

## Abstract

**Background:**

Pre-hospital ultrasound is a new challenge and lung ultrasound could be an interesting opportunity in the pre-hospital medical service. The aim of our study was to evaluate the efficacy of lung ultrasound in out-of-hospital non-traumatic respiratory insufficiency.

**Methods:**

We planned a case-controlled study in the ULSS 5 ovest vicentino area (Vicenza—Italy) enrolling subjects with severe dyspnea caused by cardiac heart failure or acute exacerbation of chronic obstructive pulmonary disease. We compared drugs administration, oxygen delivery, and laboratory tests between those patients with ultrasound integrated management and those without ultrasound.

**Results:**

Pre-hospital lung ultrasound had a high specificity (94.4%) and sensitivity (100%) for the correct identification of alveolar interstitial syndrome using B lines, whereas the percentages obtained with pleural effusion were lower (83.3, 53.3%, respectively). The patients with ultrasound integrated management received a more appropriate pharmacological therapy (*p* 0.01), as well as non-invasive ventilation (CPAP) was used more frequently in those with an acute exacerbation of chronic obstructive pulmonary disease (*p* 0.011). Laboratory tests and blood gases analysis were not significant different between the two study groups. In a sub-analysis of the patients with an A profile, we observed a significant lower concentration of PCO_2_ in those with an ultrasound integrated management (PCO_2_: 42.62 vs 52.23 *p* 0.049). According with physicians’ opinion, pre-hospital lung ultrasound gave important information or changed the therapy in the 42.3% of cases, whereas it just confirmed physical examination in the 67.7% of cases.

**Conclusions:**

Pre-hospital lung ultrasound is easy and feasible, and learning curve is rapid. Our study suggests that cardiac heart failure and acute exacerbation of chronic obstructive pulmonary disease can be considered two indications for pre-hospital ultrasound, and can improve the management of patient with acute respiratory insufficiency.

## Background

Pre-hospital ultrasound is considered one of the top five research priorities according to the opinion of a consensus meeting of a European expert panel, to identify which ultrasound examinations can be reliably transferred to the pre-hospital setting, how they affect patient management and pathway, and how providers can achieve and maintain specific ultrasound skills [1].

The indications are few and well defined, physicians and operators must be adequately trained, and the examinations must be short, focused, and strictly connected with the patients’ symptoms and history. The technical skills are the same of in-hospital critical ultrasound or even simpler [2–8].

The FAST protocol has been used successfully for the management of trauma, both inside and outside the hospital [9, 10].

Ultrasound was included in the latest guidelines for cardiopulmonary resuscitation as a supplementary tool for the identification of reversible causes of cardiac arrest [11].

In the same document, ultrasound was recognized as an additional device for airway management and for post-procedural check of the correct placement of an endo-tracheal tube [11, 12].

The lung ultrasound has changed significantly in-hospital management of non-traumatic respiratory insufficiency, and it could be a new and interesting opportunity also in the pre-hospital setting with a few and clear indications: the determination of the lung profile (A: no signs of increased extravascular lung water—“dry lung”; B: increased extravascular lung water—“wet lung”), the identification of pleural effusions, and the diagnosis of pneumothorax [2, 13].

Lung evaluation is rapid and physicians can reach a reliable diagnosis in a few minutes [13–15].

A rapid two-point technique (upper anterior and basal lateral areas) is usually enough to rule in or rule out an interstitial syndrome, a pneumothorax, or a pleural effusion [13, 15–17].

Nevertheless, there are not many studies evaluating the real effectiveness of the pre-hospital lung ultrasound: Neesse et al. [15] showed that the identification of a pleural effusion is useful for the differentiation between a cardiac heart failure (CHF) and an acute exacerbation of a chronic obstructive pulmonary disease (COPD). Strnad et al. and Ferrari and collaborators suggested that lung ultrasound is reliable to monitor response to pre-hospital treatment with continuous positive airway pressure (CPAP) in patients with CHF [18, 19].

The primary and secondary outcomes of our work evaluated the efficacy of lung ultrasound in out-of-hospital non-traumatic respiratory insufficiency, by analyzing clinical and laboratory parameters after the arrival at the emergency department. In particular, we compared drug administration, ventilation, oxygen delivery, and laboratory tests between those patients with ultrasound integrated management and those without ultrasound.

## Methods

We planned a case-controlled study in the pre-hospital emergency setting of the ULSS 5 ovest vicentino area (Vicenza—Italy) between January 2016 and December 2016.

This area included one major emergency department with 35.000 accesses par year, and a minor emergency department located in the countryside with 6000 accesses par year, each one with an emergency physician-led advanced life support (ALS) ambulance.

The former had an ultrasound portable device (Nanomax Sonosite) in the ambulance; the latter did not. All controlled cases were collected from the second one.

All patients included in the studied were treated in the major emergency department.

Inclusion criteria was severe dyspnea as prevalent symptom most likely caused by CHF or COPD exacerbation and digital pulse oximetry ≤ 90%, whereas exclusion criteria were other causes of respiratory insufficiency (for example: trauma, pulmonary embolism, cancer) and subjects less that 18 years old.

The two groups were matched for age, gender, type of respiratory insufficiency, and digital pulse oximetry recorded by the ambulance crew.

All recruited patients underwent a rapid and complete physical examination. Blood pressure, heart frequency, and pulse oximetry were tested.

Oxygen was delivered using an oxymask and inspiratory fraction of oxygen was extrapolated by oxygen flow according with the operating instructions.

Non-invasive ventilation was performed using a Boussignac system that allowed the application of a continuous positive airway pressure (PEEP) from 5 to 10 cmH_2_O and the possibility to choose among three different percentages of oxygen: 30, 50, and 100%. The criteria used for using the CPAP were the presence of at least one of the following findings: a persistent oximetry lower than 90% even after oxygen supplementation with the oxymask, respiratory rate higher than 30 per min, mild drowsiness.

Lung ultrasound was performed after the clinical examination with a rapid two-point technique (upper anterior and basal lateral areas). The type of lung profile (A profile: dry lung; B profile: wet lung) and the presence of a pleural effusion were recorded.

An interstitial syndrome was defined by the presence of three or more B lines in a longitudinal plane between two ribs in two or more regions bilaterally with a symmetrical pattern, with or without a pleural effusion.

All ultrasound examinations were performed with the same ultrasound device (Sonosite Nanomax) using a convex probe (3.5–5 MHz). A linear probe was used to improve the accuracy in case of an undefined diagnosis.

Operators were certified emergency physicians who had accomplished a full mentoring program for “UltraSound Life Support”.

In-hospital assessment included a physical examination, laboratory tests, blood gases analysis, a chest X-ray, in-hospital lung ultrasound, a focused echocardiography, and a static and dynamic evaluation of the vena cava.

The diagnosis made after discharge from the emergency department or after hospitalization was compared with the one made by the physician in the ambulance.

In-hospital lung ultrasound was blinded with respect to pre-hospital one and performed soon after the arrival at the hospital by the emergency physician of the emergency department.

The study evaluated the feasibility of pre-hospital lung ultrasound and the improvement of both pharmacological and oxygen administration in the ambulance and of the blood gases analysis (pH and CO_2_) at the arrival at the emergency department.

Finally, we compared hospitalization rate and the time spent in the emergency department between the two study groups.

The study was conducted in accordance with Helsinki Declaration, it had been previously approved by local medical ethics committee and a written informed consent was collected from all the patients.

### Statistical analysis

We expressed as mean and standard deviation all the anthropometric and laboratory parameters of the patients.

Standard *T* test was used for the comparison of parametric variables, whereas Chi-square analysis was chosen for non-parametric data.

Sensitivity, specificity, and positive (PPV) and negative (NPV) predictive values were calculated to test the diagnostic capability of the pre-hospital lung ultrasound.

Finally physician’s opinions about the utility of the pre-hospital ultrasound were transformed into percentages.

Results were considered statistically significant for *p* value lower than 0.05.

Statistical analysis was made using SPSS 16.0.

## Results

We recruited 30 patients affected by non-traumatic respiratory insufficiency caused by CHF (12 patients) or acute exacerbation of COPD (18 patients), who underwent an ultrasound integrated management (US group) and were compared with 30 patients managed without ultrasound (NUS group).

The characteristics of the two groups are described in Table 1. The two groups were comparable for age, gender, type of respiratory insufficiency, and pulse oximetry values at the arrival of the ALS ambulance.

**Table 1:** General characteristics of the two study groups

	Mean NUS	SD NUS	Mean US	SD US	*p*
Age	83.87	± 10.08	80.30	± 12.27	ns
Hb	123.93	± 18.90	125.35	± 25.72	ns
WBC	14.33	± 15.59	12.56	± 5.16	ns
Cr	1.47	± 1.35	1.29	± 1.76	ns
CRP	91.70	± 92.69	75.08	± 73.46	ns
pH	7.36	± 0.09	7.38	± 0.08	ns
PCO_2_	49.32	± 16.87	44.36	± 11.69	ns
SBP	144.20	± 38.27	142.58	± 33.48	ns
DBP	86.43	± 16.70	79.72	± 22.73	ns
HR	102.89	± 27.83	104.72	± 21.94	ns
O_2_%	82.20	± 7.81	79.43	± 11.83	ns

*Hb* haemoglobin (g/L), *WBC* white blood cells (x 10^9^ L), *Cr* creatinine (mg/dL), *CRP* C reactive protein, *PCO*_*2*_ partial pressure of carbon dioxide, *SBP* systolic blood pressure (mmHg), *DBP* diastolic blood pressure (mmHg), *HR* heart rate, *O*_*2*_*%* pulse oximetry

Pre-hospital lung ultrasound was accurate for the identification of the correct lung profile, in particular for the diagnosis of an interstitial syndrome.

B lines had both a high sensitivity (100%) and specificity (94.4%), and a high PPV and NPV (92.3 and 100%, respectively) for the diagnosis of cardiac heart failure.

The presence of a pleural effusion was not as accurate as B lines for CHF: sensitivity 83.3%, specificity 58.3%, PPV 75.0%, and NPV 70.0%.

Finally, the same percentages obtained by the combination of B lines with pleural effusion for the diagnosis of CHD were 77.8, 100, 100, and 75.5%, respectively.

**Fig. 1:**
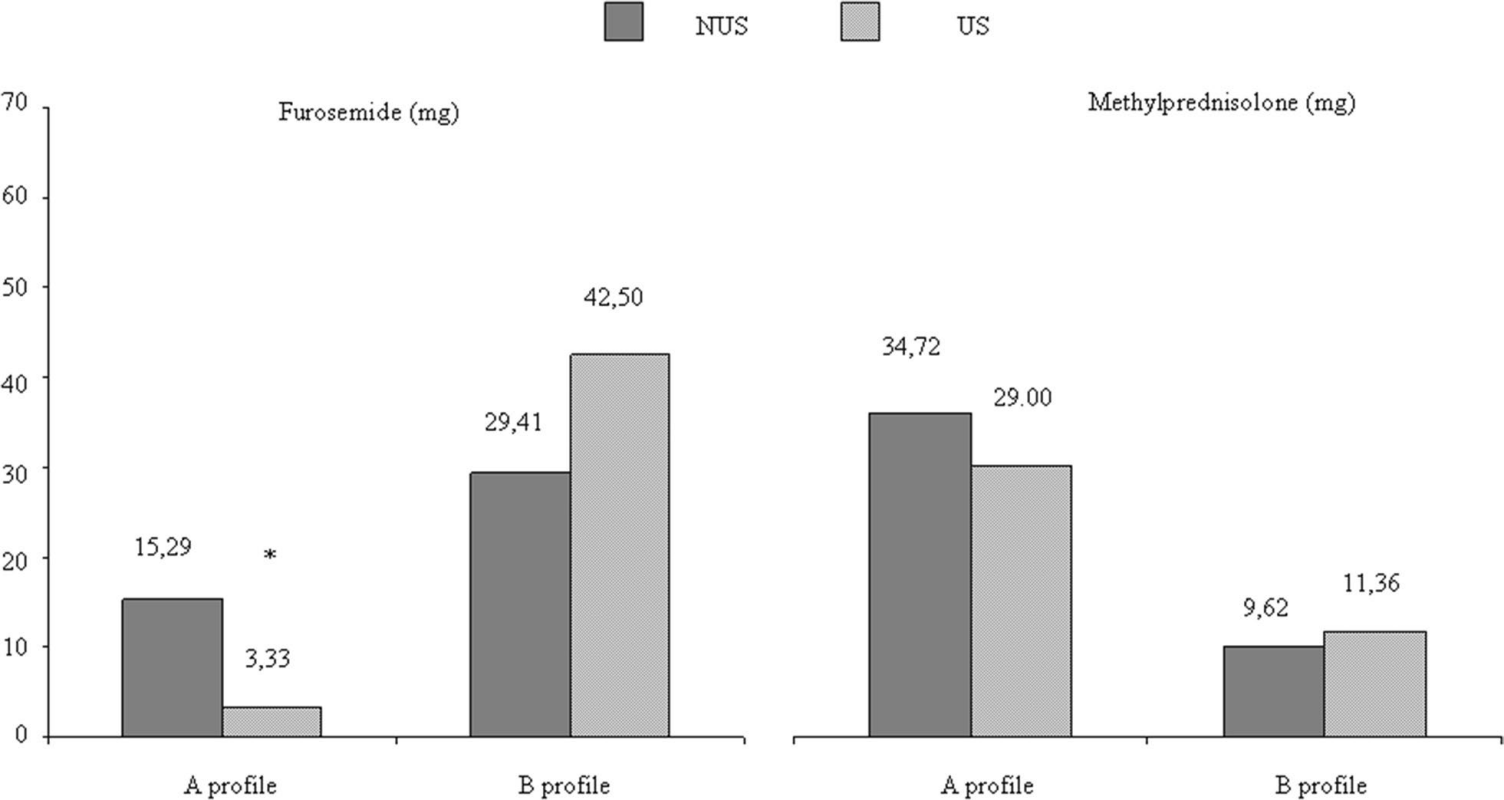
Pharmacological therapy in the two study groups splitted by lung profile

The number of patients who received an appropriate pharmacological treatment was significantly higher in US group (*p* < 0.001), especially in those subjects with an A profile (non-cardiogenic respiratory insufficiency—COPD; *p* < 0.0001). In this subgroup, the mean dose of furosemide was significantly lower in the US group than the NUS group (3.33 mg vs 15.29 mg *p* = 0.036). On the contrary, in the subgroup of patients with a B profile (CHF), the mean dosage of furosemide was higher in the US groups (42.50 mg vs 29.41 mg), even though not significantly (Fig. 1).

The corticosteroid administration (methylprednisolone) was comparable both between the two groups with an A profile (34.72 vs 29.00 mg) and with a B profile (9.62 vs 11.36 mg) (Fig. 1).

We did not find any difference in the pre-hospital administration of other medications such us morphine, salbutamol, nitroglycerin, and vasoactive agents.

Comparing the use of CPAP, in those patients with an A profile, it was employed more in the US group than in the NUS one (*p* 0.011), whereas the use was comparable in the two groups with a B profile. Moreover, the FIO_2_ administered was not significantly different between groups.

Blood gases analysis was not significantly different in the US group than in the NUS group.

**Fig. 2:**
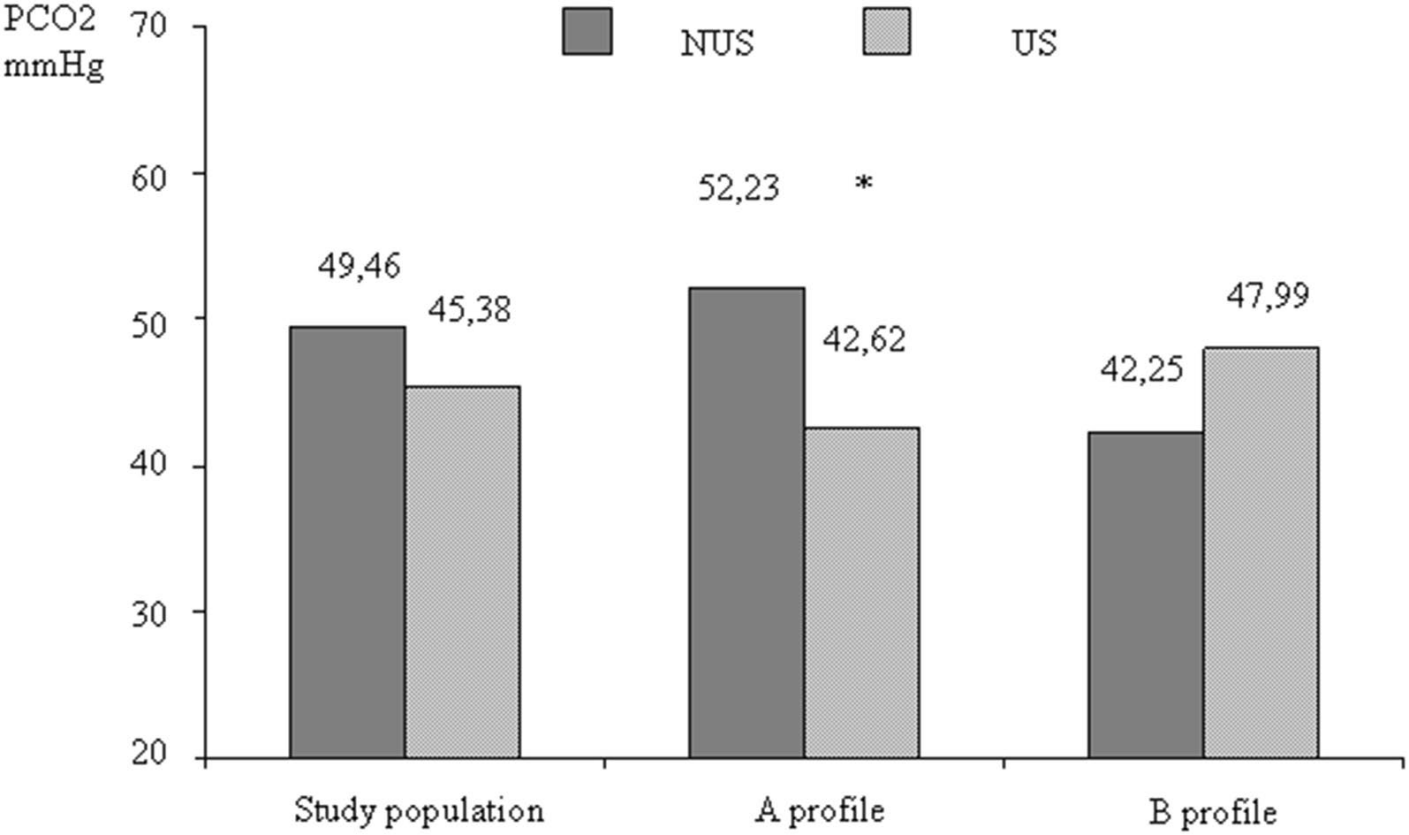
PCO_2_ concentration in the two study groups splitted by lung profile

Anyway, a sub-analysis of patients with the A profile showed that PCO_2_ was significantly lower in the US group than NUS group (PCO_2_: 42.62 vs 52.23 *p* 0.049) (Fig. 2).

We did not found any significant difference in all the other laboratory examinations (Table 1).

Two patients were intubated in the NUS group and one in the US group, without any statistical significance.

The convex probe was always the probe of choice and the linear probe was never used.

Hospitalization rate was comparable between the two study groups, whereas we observed a reduction of the overall time spent in the emergency department by the US group (267.77 ± 406.15 vs 463.24 ± 521.29 min), but these data did not reach a full statistical significance.

According with physicians’ opinion, pre-hospital lung ultrasound either gave important information or changed the therapy in the 42.3% of cases, whereas it just confirmed physical examination and therapy in the remaining 67.7% of cases.

## Discussion

Lung ultrasound is undoubtedly an effective and reliable instrument capable to improve medical care [13].

It has changed in-hospital management of respiratory insufficiency, it has demonstrated remarkable results in patients with trauma of the chest, and both the FAST and the Extended-FAST (EFAST) are two effective protocols that are used worldwide [9, 10, 20–23].

Anyway it is not clear if the same efficacy can be exploited in the pre-hospital medical service for non-traumatic respiratory insufficiency [15–18].

The critical points are three: which are the most remarkable indications? Does it influence the management of respiratory insufficiency? Does it change short- and long-term prognosis?

In our article, we focused our attention on patients affected by cardiac heart failure and acute exacerbation of chronic obstructive pulmonary disease.

**Fig. 3:**
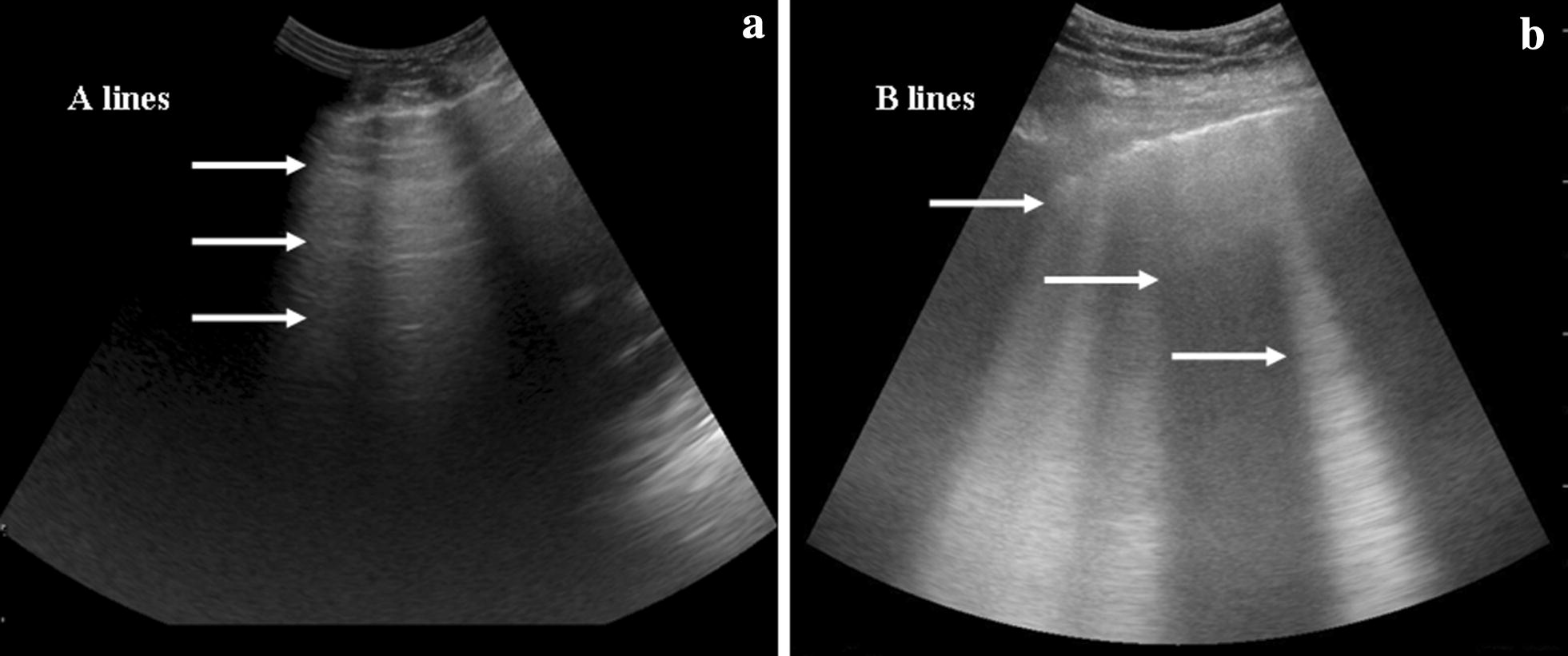
**a** A profile—dry lung; **b** B profile—wet lung

We chose CHF and COPD, because their ultrasound patterns are completely different and ultrasound has a high sensitivity and specificity for the identification of the type of lung profile: the former has a B profile (wet lung) and the latter an A profile (dry lung) (Fig. 3) [13, 24].

Our data confirm that pre-hospital ultrasound can differentiate them with a high sensitivity and sensibility.

The examination time was very rapid, all the scans lasted less than 3 min, the two points technique was adequate to examine the chest and the convex probe was always appropriate [2, 13].

The linear probe would have been used if lung profile had not been clear enough, but it never happened.

We tested B lines, the pleural effusion, and the combination between them as ultrasonographic signs for the diagnosis of CHD.

Neesse et al. [15] showed that the identification of a pleural effusion is useful for the diagnosis of CHD in the pre-hospital setting. Nevertheless, our data indicate that B lines had a higher sensitivity and specificity and the combination of the two findings did not improve the diagnostic capability.

Since the identification of B lines is simpler and faster than the diagnosis of pleural effusion, we think that the presence of a bilateral, diffuse, and symmetrical B profile of the lungs is enough for the diagnosis of CHD in a pre-hospital setting.

We did not have any recordings about the time required for the sole identification of the lung profile, but it would have been certainly shorter than the 3 min described above, probably within 1 min.

The second question regards the possibility to influence medical decision-making?

A more accurate diagnosis brought about a more adequate therapy.

In the US group, the patients with CHF received a higher dosage of furosemide, even though not significantly, whereas it was significantly lower in case of COPD. The data are certainly more remarkable in those with an acute exacerbation of COPD, because it reduced inappropriate drug administration preventing hypotension, dehydration, and electrolyte imbalance.

Non-invasive ventilation (CPAP) was used thoroughly in patients affected by CHD, whereas, in those with COPD exacerbation, it was applied more often in the US group than in the NUS group. We think that the possibility to identify the correct lung profile made the physicians more confident in choosing the most appropriate type of ventilation and setting for the CPAP.

Eventually in-hospital blood gas analysis showed a lower level of PCO_2_ in patients affected by COPD when ultrasound was used. The better diagnostic performances given by ultrasound helped the physicians to titrate the oxygen better, reducing the risk of hypercapnia and acidosis.

One of the most remarkable consideration that comes out from these results is that ultrasound made more homogeneous the pre-hospital approach, independently from the medical specialization of the physicians. Indeed, since the physicians who worked in the ambulance with the ultrasound and in the one without were the same, we might assess that the improvement of diagnostic accuracy and medical assistance was determined by the ultrasound integrated approach.

This was possible, because the identification of the lung patter is simple and extremely different between a pulmonary oedema and an acute exacerbation of COPD (Fig. 3). The possibility to “watch the diagnosis on the screen” permitted to choose easily the most appropriate therapy, despite all the confounding elements that are usually caused by the noise and the narrow spaces inside the ambulance or by the patient conditions.

Moreover, the learning curve for the identification of the lung profile is easy and rapid [25]. Consequently, it lets also paramedics, who work in the pre-hospital medical service, to approach lung ultrasound, as it has already been done with FAST, so that the choice of ventilation, oxygen administration, and CPAP setting would become easier and safer for everyone.

Finally, we are not able to answer the third question: we did not collect enough information about the prognosis and the outcome of patients. Anyway, we found a reduction of the time spent in the ED by the patients with the ultrasound integrated approach. We think that the result is interesting even if it was not completely significant and, according to the extent of the reduction, the lack of significance might be caused by the small number of subjects. The possibility to reach the correct diagnosis and to set the right therapy earlier permitted to speed up the in-hospital management of the patients.

## Limitations

The study has certainly several limitations.

First of all, the number of patients is low and should be increased, including other causes of respiratory insufficiency.

Then, it is a case–control study, whereas a randomized-controlled trial could have been more adequate from a statistical point of view.

Most of pre-hospital scans were not re-evaluated by a blinded second operator, because they had not been stored or recorded properly.

The two study groups were matched by comparing digital oxygen values recorded by the ambulance crew, without considering the duration of symptoms and previous history of COPD.

## Conclusions

Pre-hospital lung ultrasound is easy and feasible, and learning curve is very rapid [25].

Pulmonary oedema and COPD should be considered as proven indications for pre-hospital ultrasound, since it can improve both pharmacological therapy and oxygen delivery.

Our study population was small, so that other studies should be planned to support our findings, and to establish the influence of ultrasound on invasive management of airways and on short- and long-term prognosis.

## Abbreviations

ALS: advanced life support
COPD: chronic obstructive pulmonary disease
CHF: cardiac heart failure
CPAP: continuous positive airway pressure
Cr: serum creatinine
CPR: C reactive protein
DBP: diastolic blood pressure
FAST: fast assessment sonography for trauma
EFAST: extended fast assessment sonography for trauma
FIO_2_: fraction of inspired oxygen
Hb: haemoglobin concentration
HR: heart rate
LUS: lung ultrasound
NUS group: group without an ultrasound integrated management
PCO_2_: partial pressure of carbon dioxide
PEEP: continuous positive airway pressure
PPV: positive predictive value
NPV: negative predictive value
O_2_%: pulse oximetry
SBP: systolic blood pressure
US group: ultrasound integrated management group
WBC: white blood cells
